# Removal of a Tumor Imbedded in the Parotid Gland

**Published:** 1852-01

**Authors:** 


ARTICLE XIV.

*Removal of a Tumor imbedded in the Parotid Gland.*

MR. CAUTON related the following particulars of a case which came under his hands before the Westminster Medical Society.

A man, aged sixty, had for several years been afflicted with amaurosis of both eyes, for which mercury, strychnine, galvanism, and other remedies, had been ineffectually employed. Some time since he complained of a swelling below the left ear, and which had gradually increased in size. It gave no pain; but by degrees the resulting inconveniences were, difficulty in mastication, deglutition, and great impairment of hearing. The tumor was firmly wedged in between the ramus of the jaw and the mastoid process, extending chiefly towards the angle of the former. The skin over it was healthy and unadherent, and to the touch it was firm and resisting. *Operation:* The superficial incision was commenced at the zygoma, extended thence, in a curved direction, along the anterior boundary of the tumor, and terminated a little way behind the angle of the jaw. The flap being dissected backwards, the tumor was seized by a double hook ; and as traction was therewith made upon it, the knife isolated it from its connections. A portion of the surrounding parotid gland required to be likewise removed. The depth to which the tumor extended was greater than the extent of its growth superficially ; and on removal, it was seen to be an enlarged and apparently fungoid state of a lymphatic gland, inclosed in a strong, fibrous-looking capsule. The hemorrhage was trivial, and the patient recovered without any paralysis of the face, and with restoration of the power of mastication, deglutition and hearing.

